# Functional Connectivity of the Nucleus Accumbens across Variants of Callous-Unemotional Traits: A Resting-State fMRI Study in Children and Adolescents

**DOI:** 10.1007/s10802-023-01143-z

**Published:** 2023-10-25

**Authors:** Jules Roger Dugré, Stéphane Potvin

**Affiliations:** 1 School of Psychology and Centre for Human Brain Health, University of Birmingham, Birmingham, B15 2TT England; 2 Research Center of the Institut Universitaire en Santé Mentale de Montréal, Hochelaga, Montreal, 7331, H1N 3V2 Canada; 3 Department of Psychiatry and Addictology, Faculty of medicine, University of Montreal, Montreal, Canada

**Keywords:** Nucleus Accumbens, Functional Connectivity, Resting-State, Callous-Unemotional Traits, Anxiety

## Abstract

A large body of literature suggests that the primary (high callousness-unemotional traits [CU] and low anxiety) and secondary (high CU traits and anxiety) variants of psychopathy significantly differ in terms of their clinical profiles. However, little is known about their neurobiological differences. While few studies showed that variants differ in brain activity during fear processing, it remains unknown whether they also show atypical functioning in motivational and reward system. Latent Profile Analysis (LPA) was conducted on a large sample of adolescents (n = 1416) to identify variants based on their levels of callousness and anxiety. Seed-to-voxel connectivity analysis was subsequently performed on resting-state fMRI data to compare connectivity patterns of the nucleus accumbens across subgroups. LPA failed to identify the primary variant when using total score of CU traits. Using a family-wise cluster correction, groups did not differ on functional connectivity. However, at an uncorrected threshold the secondary variant showed distinct functional connectivity between the nucleus accumbens and posterior insula, lateral orbitofrontal cortex, supplementary motor area, and parietal regions. Secondary LPA analysis using only the callousness subscale successfully distinguish both variants. Group differences replicated results of deficits in functional connectivity between the nucleus accumbens and posterior insula and supplementary motor area, but additionally showed effect in the superior temporal gyrus which was specific to the primary variant. The current study supports the importance of examining the neurobiological markers across subgroups of adolescents at risk for conduct problems to precise our understanding of this heterogeneous population.

**Supplementary Information:**

The online version contains supplementary material available at 10.1007/s10802-023-01143-z.

## Introduction

Research has shown that individuals with high psychopathic traits form a heterogeneous population. For instance, Karpman postulated the presence of two clinical entities, both characterized by high levels of psychopathic traits but distinct levels of emotional lability, suggesting that psychopathy may result from multiple etiological pathways (Karpman, 1941). More recently, this distinction has been extended to children with high callous-unemotional traits (CU) with and/without severe levels of anxiety. Indeed, children with the primary variant (i.e., high CU traits and low anxiety) are thought to display low emotional arousal and a hypo-reactivity to social cues (e.g., facial expression of fear), whereas emotional hyperarousal and high sensitivity to negative emotions may represent the core features of the secondary variant (i.e., high CU traits and anxiety) (Craig et al., 2021). In their recent literature review, Craig et al. (2021) showed that 83.3% of the included studies reported that the secondary variants had significantly higher levels of childhood adversities (e.g., abuse, traumas) compared to their counterparts in the primary variant and controls. There is also evidence that children with the secondary variant also show more severe hyperactivity/impulsivity traits, internalizing traits, irritability, aggressive behaviors, substance misuse and suicidal behaviors (Cecil et al., 2018; Fanti et al., 2013; Goulter et al., 2017; Huang et al., 2020; Kimonis et al., 2012; Meehan et al., 2017). Although studies found significant differences between variants at a clinical level, the neurobiological markers of these variants remain largely understudied. Nevertheless, it has been postulated that variants of CU traits (and psychopathy in adults) may mainly differ in amygdala reactivity during fear processing, given that psychopathic traits and anxiety are linked to opposite activity in such region (decreased and increased, respectively) (Ashworth et al., 2021; Blackford & Pine, 2012; Dugré et al., 2020; Poeppl et al., 2019). Indeed, recent studies showed consistent differences between variants in the amygdala (as a predefined region-of-interest) during fear processing (Fanti et al., 2020; Meffert et al., 2018; Sethi et al., 2018). Moreover, Motzkin and colleagues (2011) showed that the functional connectivity between the amygdala and the ventromedial prefrontal cortex differentiated both variants in adults. Aside from the interests for fear processing, the differences between variants regarding the neurobiological mechanisms underpinning motivation, reward processing and decision-making remains largely understudied.

Preclinical research pursued in the last decades has provided substantial evidence that the dopaminergic neurons projecting from the ventral tegmental area to the nucleus accumbens (NAcc) / ventral striatum (VS) and the ventro-medial prefrontal cortex (vmPFC) play a key role in motivation (Haber & Knutson, 2010; Wise, 2002). Coherently with these findings, past meta-analyses of functional neuroimaging studies in humans have consistently showed that the NAcc is involved in reward processing (Diekhof et al., 2012; Flannery et al., 2020; Liu et al., 2011; Sescousse et al., 2013), subjective valuation (Bartra et al., 2013; Clithero & Rangel, 2014) and reward prediction error (Corlett et al., 2022). In adults, the VS shows positive connectivity with the medial prefrontal cortex (including the ventromedial prefrontal cortex and orbitofrontal cortex), subcortical structures (e.g., amygdala and hippocampus), posterior cingulate cortex and insular cortex (i.e., anterior to posterior), and negative connectivity with the anterior midcingulate cortex, the supplementary motor area, the superior temporal gyrus and superior parietal lobule (Di Martino et al., 2008; Janssen et al., 2015; Zhang et al., 2017). From childhood to adulthood, the resting-state functional connectivity between the NAcc and frontal regions (including perigenual and subgenual anterior cingulate cortex, ventromedial prefrontal cortex, and orbitofrontal cortex) linearly decreases, whereas its connectivity with the posterior insula shows a quadratic effect (Fareri et al., 2015), highlighting its potential role in the development of various psychopathologies. In fact, a growing body of literature show that functional connectivity of the NAcc is associated with numerous psychopathologies during adolescence such as anxiety and depressive symptoms (Dorfman et al., 2016; Pan et al., 2017), impulsive decision-making (Costa Dias et al., 2013), substance misuse (Huntley et al., 2020; Morales et al., 2021), and social problems (Fareri et al., 2017). Thus, the maturational deficits in motivational processes are thought play a major role in our understanding of externalizing problems in children and adolescents (Bjork & Pardini, 2015).

Prior work has shown that adolescents with CU traits (Blair et al., 2001; Scerbo et al., 1990) and adults with psychopathic traits (Blair et al., 2006; Mitchell et al., 2002; Newman & Kosson, 1986) may exhibit atypical reward processing, that is, they are more likely to persist in a previously rewarded response even when the risk for punishment/losses increase. In the neuroimaging literature, the effect of CU traits on brain activity during reward fMRI tasks yields inconsistent results across studies (Byrd et al., 2014; Murray et al., 2018). For example, in a community sample of healthy adolescents, CU traits correlated with activity of the VS during reward anticipation, but the effect was no longer significant when controlling for severity of externalizing problems (Huang et al., 2019). Some have found that CU traits were negatively associated with the medial prefrontal cortex but not the ventral striatum during reward anticipation (Veroude et al., 2016), whereas others found that CU traits were unrelated to reward anticipation (Murray et al., 2023). When receiving rewards, youths with disruptive behavior disorder (DBD) and elevated CU traits showed reduced activity of the dorsal striatum (but not ventral) as a function of prediction error when receiving reward (White et al., 2013). Similarly, Zhang and colleagues (Zhang et al., 2023) found that CU traits were negatively associated with activity of the dorsal striatum (but not the ventral part) in response to reward relative to punishment. These conflicting results may be partially explained by the relatively small sample sizes used to detect significant effect of the ventral striatum. For example, in a recent study of 995 youths with DBDs, Hawes and colleagues (Hawes et al., 2021) found that those with high CU traits (DBD + CU) were characterized by reduced activity in dorsal anterior cingulate cortex (as well as those with low CU traits [DBD-CU]) compared to their counterparts in the typically developing group during reward anticipation. Children with DBD-CU additionally exhibited reduced ventral and dorsal striatal activity during reward anticipation (Hawes et al., 2021). When receiving rewards, both DBD + CU and DBD-CU groups showed greater activation of the NAcc and OFC, compared to controls (Hawes et al., 2021). Other studies found limited evidence of differences in brain activity during reception of reward between children with high CU traits and high conduct problems and controls (Byrd et al., 2018; Finger et al., 2011). In adults, some studies showed that the severity of psychopathic traits correlated with VS activity when anticipating rewards (Bjork et al., 2012), whereas others showed no such effect when viewing drug cues (Cope et al., 2014) or a greater effect in loss rather reward reception (Pujara et al., 2014). Across the limited number of studies using the NAcc (or VS) as a seed of interest during resting-state, similar divergence across results is observed. Indeed, Hosking et al. (Hosking et al., 2017) found that the functional connectivity between the NAcc and the medial prefrontal cortex was negatively associated with severity of psychopathic traits in incarcerated adults (PCL-R). Moreover, Factor 2 of the PCL-R (but not Factor 1) positively correlated with functional connectivity between the NAcc and dorsolateral prefrontal cortex and negatively correlated with functional connectivity between the NAcc and the postcentral gyrus (Korponay et al., 2017). However, other studies found no significant difference in VS functional connectivity between adult offenders with psychopathy and those without psychopathy (Motzkin et al., 2014) or between adults with an antisocial personality disorder (ASPD) and elevated psychopathic traits (PCL-R ≈ 25) and those without ASPD (Kolla et al., 2018).

It is noteworthy to mention that these discrepancies may principally originate from the large heterogeneity in population with high CU/psychopathic traits (e.g., variants). Indeed, recent results indicate that at low levels of social adversities (e.g., Foster Home, Divorced Parents, Welfare Food Stamps), high CU traits were associated with reward hypo-responsivity (i.e., less pre-ejection period shortening), whereas higher CU traits were associated with reward hyper-responsivity at high levels of social adversities (Gao & Zhang, 2021). In addition, some preliminary results also suggest that individuals with the primary variant (but not those on the secondary variant) may be unable to integrate socio-affective information into decision-making to select the appropriate behaviors (Koenigs et al., 2010, 2012). While adolescents with high CU traits and adults with high psychopathic traits may show aberrant reward processing and decision-making, the neurobiological differences between the primary variant (hypo-arousal) and those with the secondary variant (hyper-arousal) remain to be elucidate.

To our knowledge, no studies have examined the NAcc functional connectivity between variants of CU traits, leaving unknown whether they may be characterized by specific neurobiological impairments. Despite that variants are well described at a clinical level, searching for neurobiological markers of variants in childhood and adolescence is of utmost importance to gain insight of their underlying mechanisms and better characterize their developmental route. To achieve this goal, we conducted a latent profile analysis (LPA) to extract data-driven subgroups in a large sample of children and adolescents using callousness and anxiety as dimensions of interests. We subsequently conducted seed-to-voxel analyses using the bilateral NAcc as seeds of interest to examine differences in functional connectivity between variants. Given that some evidence suggests that primary variant (but not the secondary variant) may show similar utilitarian decision-making and clinical presentation as patients with lesions to the vmPFC (Koenigs et al., 2010), we further hypothesized that this hypo-arousal group may be characterized by decreased functional connectivity within the mesocorticolimbic system (i.e., NAcc and vmPFC, as similarly found in (Hosking et al., 2017), whereas the secondary variant (hyper-arousal group) may rather be characterized by decreased connectivity between the NAcc and regions involved in regulatory mechanisms (e.g., ventro- and dorso-lateral PFC, aMCC/pre-SMA, see meta-analyses on emotion regulation: (Kohn et al., 2014; Zilverstand et al., 2017) given their potential hyper-responsivity to reward (Gao & Zhang, 2021). In addition, considering that some effects found in reward processing are also related to severity of impulsivity/antisocial factor and are observed in adolescents and adults with Conduct Disorder/Antisocial Personality Disorder (Buckholtz et al., 2010; Carré et al., 2013; Hawes et al., 2021; Huang et al., 2019; Murray et al., 2018; Rubia et al., 2009) (Bubenzer-Busch et al., 2016; Crowley et al., 2010; Völlm et al., 2007), we conducted supplemental analyses controlling for the severity of hyperactivity/impulsivity symptoms as well as conduct problems.

## Methods and Materials

### Participants

Data from 2200 participants were obtained from the Healthy Brain Network (HBN), an ongoing initiative in New York area (USA) that aims to investigate heterogeneity and impairment in developmental psychopathology (5–21 years old) (Alexander et al., 2017). The HBN adopted a community-referred recruitment model in which advertisements was provided to community members, educators, parents. Exclusion criteria were impairments that prevents full participation in the study (e.g., serious neurological disorders, hearing or visual impairments), neurodegenerative disorder, acute encephalopathy, acute intoxication, and serious psychiatric disorders (recent diagnosis of schizophrenia and/or manic episode). Supplemental information is provided elsewhere (Alexander et al., 2017).

From the 2200 participants included in the Data Release 7.0, 1583 participants contained available functional neuroimaging data. Written assent was obtained from participants younger than 18 years old, and written consent was obtained from their legal guardians. Written informed consent was obtained from participants aged 18 or older prior to enrolling in the study. The original HBN study was approved by the Chesapeake Institutional Review Board (now Advarra Inc., see https://www.advarra.com/). The current study was approved by the local ethics committee (Centre Intégré Universitaire de Santé et de Services Sociaux de l’Est-de-l’Île-de-Montréal).

### Assessments

Severity of callousness was evaluated using the well-validated parent-report Inventory of Callous-Unemotional Traits (Total score, Cronbach alpha, α = .88). (Frick, 2004; Wang et al., 2017). We also focused on the Callousness subscale which includes 11 items rated on a 4-point scale (0 = not true at all to 3 = definitively true) such as “*Seem very cold and uncaring to others*” and “*Does not care who he/she hurts to get what he/she wants*” (Cronbach alpha, α = .765). First, it is noteworthy to mention that CU is a umbrella term referring to correlated, yet distinct subconstructs such as callousness (e.g., “*I do not care who I hurt to get what I want*”), uncaring (e.g., *I always try my best*”, “*I work hard on everything I do*”) and unemotionality (e.g., “*I do not show my emotions to others*”) traits (Frick, 2004). Indeed, results from a recent meta-analysis showed that callousness is only moderately correlated with uncaring (pooled r = 0.45) and weakly correlated with unemotional traits (pooled r = 0.24). Second, the callousness subscale showed greater association with the affective facet of psychopathic traits, internalizing and externalizing problems, compared to other subconstructs (Cardinale & Marsh, 2020). These findings support the importance of delineating between CU subconstructs when studying inter-individual variations. Third, evidence suggests that the callousness is the most discriminatory subscale between variants and controls (Kimonis et al., 2017a, b; Pechorro et al., 2022). This could be potentially explained by the fact that callousness seems to be the only subconstruct associated with levels of internalizing symptoms (Cardinale & Marsh, 2020) including anxiety (Kimonis et al., 2013), indicating potential differences in the underlying mechanisms of CU subconstructs. Finally, neuroimaging studies support distinct associations between CU subconstructs (specifically callousness) and brain activity/functional connectivity measures (Lockwood et al., 2013; Werhahn et al., 2021; Yoder et al., 2016).

Anxiety was assessed using the total score of the parent-report Screen for Child Anxiety Related Disorders (SCARED) (Birmaher et al., 1999). The SCARED is constituted by 41 items rated using a 3-point scale (0 = not true/hardly ever true to 2 = very true/often true). This scale showed good internal consistency (α = 0.927).

Conduct problems were assessed using the Child Behavior Checklist (CBCL, (Achenbach & Rescorla, 2001), which comprised 33 items from Aggressive (20 items) and Rule-Breaking (11 items) syndromes scales. Parents rated each item using a 3-point scale (0 = not true to 2 = very true)(α = 0.926). In the current study, the standardized conduct problems score was used (T-score). We also examined the confounding effects of ADHD symptoms (i.e., hyperactivity/impulsivity and inattention) using the Strengths and Weaknesses of ADHD and Normal Behavior Rating Scale (SWAN)(Swanson et al., 2012). Parents rated child’s behaviors (18 items) on a 7-point likert scale (-3 = far above average to 3-far below average). Finally, negative life events experienced by children were assessed by their parents using a total count score of presence or absence of 21 events (e.g., “*suffered from serious illness*”, “*Close friend died*”, “*Parents have serious money troubles*”, “*parents lost a jobs*”) with the Negative Life Events Scale (NLES-P, α = 0.69) (Sandler et al., 1991).

### Latent Profile Analysis

Identification of subgroups based on severity of Anxiety and ICU (Total Score & Callousness) was performed using Latent Profile Analysis (LPA) in MPLUS 6.12 (Muthén et al., 2012). Subjects with missing data on both variables were listwise excluded. Full-information maximum likelihood (FIML) estimator under the missing at random assumption computed the parameter estimates for missing values. In contrast to use *age* as a simple covariate in LPA, we controlled for age by using the KNOWNCLASS option. More precisely, we modelled the LPA to allow differences in items variances across developmental groups but kept scales means and class probabilities fixed across age groups. Models with 2 to 5 classes were tested. Several metrics were used to evaluate the different models. Indeed, the best model was selected by identifying the elbow when plotting the Aikaike (AIC) and Bayesian (BIC) Information criteria as well as the sample-size adjusted BIC (Akaike, 1987; Schwarz, 1978). Moreover, the entropy (closest to 1.0) (Celeux & Soromenho, 1996), the Average posterior probabilities (AvePP > 0.80) (Clark & Muthén, 2009) and the smallest class size (> 1.0%) were also used as criteria. Subjects were grouped based on their highest probabilities of belongingness to a particular class (latent classes). Subgroups were subsequently compared on sociodemographic and clinical variables using Chi-squared and Kruskal-Wallis tests with Dunn-Bonferroni post hoc tests.

### MRI Data Acquisition Parameters

MRI acquisition took place at three different sites: mobile 1.5T Siemens Avanto in Staten Island, 3T Siemens Tim Trio at Rutgers University Brain Imaging Center (RUBIC), and 3T Siemens Prisma at the CitiGroup Cornell Brain Imaging Center (CBIC). Acquisition parameters for the three sites are described in Table 1. Data at the CBIC were obtained using the same data acquisition protocol implemented at RUBIC. The acquisition of the two resting-state scans lasted 5 min each, during which participants viewed a fixation cross located at the center of the computer screen. Data for the Siemens Avanto were acquired in a single run lasting 10 min. More information can be found elsewhere (Alexander et al., 2017) https://fcon_1000.projects.nitrc.org/indi/cmi_healthy_brain_network/).

**Table 1:** MRI Scan Parameters

	Slices	Resolution (mm)	TR (ms)	TE (ms)	Tl (ms)	Flip angle (deg)	Multi-band	
Staten Island (1.5T Siemens Avanto)								
T1	176	1.0 × 1.0 × 1.0	2730	1.64	1000	7	Off	
fMRI	54	2.5 × 2.5 × 2.5	1450	40	N/A	55	3	
RUBIC (3T Siemens Trio Tim)								
T1	224	0.8 × 0.8 × 0.8	2500	3.15	1060	8	Off	
fMRI	60	2.4 × 2.4 × 2.4	800	30	N/A	31	6	
CBIC (3T Siemens Prisma)								
T1	224	0.8 × 0.8 × 0.8	2500	3.15	1060	8	Off	
fMRI	60	2.4 × 2.4 × 2.4	800	30	N/A	31	6	

*Note.* RUBIC = Rutgers University Brain Imaging Center; CBIC = CitiGroup Cornell Brain Imaging Center

### fMRI Data Preprocessing

Functional images were realigned, corrected for motion artifacts with the Artifact Detection Tool (Power et al., 2014) (ART, setting a threshold of 0.9 mm subject ART’s composite motion and a global signal threshold of Z = 5) with the implemented in CONN Toolbox (Whitfield-Gabrieli & Nieto-Castanon, 2012), bandpass filtered (0.01 Hz < f < 0.10 Hz) and co-registered to the corresponding anatomical image. The anatomical images were segmented (into GM, white matter, and cerebrospinal fluid) and normalized to the Montreal Neurological Institute (MNI) stereotaxic space. Functional images were then normalized based on structural data, spatially smoothed with a 6 mm full-width-at-half-maximum (FWHM) 3D isotropic Gaussian kernel and resampled to 2 mm^3^ voxels. For the preprocessing, the anatomical component-based noise correction method (aCompCor strategy, (Behzadi et al., 2007), was employed to remove confounding effects from the BOLD time series, such as the physiological noise originating from the white matter and cerebrospinal fluid. This method was found to increase the validity and sensitivity of analyses (Chai et al., 2012). In the current study, preprocessing issues were found in 108 participants (n = 1475), and 59 adolescents exhibited high movements (exceeding 3 mm), leaving a final sample size of 1416 adolescents.

### Seed-based Connectivity Analyses

Both left and right NAcc were selected as seeds from the FSL Harvard-Oxford Atlas, provided in the CONN Toolbox (mask). Physiological noise, realignment parameters, and movement artifacts were regressed out as confounding effects from the BOLD time-series at each voxel. In the first-level analysis, Pearson’s correlation coefficients between the residual BOLD time course from each seed and the time course of all other voxels, for each subject. Coefficients were converted to normally distributed z-scores using a Fisher Z-Transformation. Second-level analyses (F-tests) were conducted to examine differences in NAcc connectivity between latent classes. We tested significant differences with a conservative threshold (p < 0.001 at a voxel level with family-wise correction [FWE] p < 0.05) as well as a more liberal threshold (p < 0.001 at a voxel level, 20 voxels extent) to balance between Type I and Type II errors, while adjusting for age, site, sex, percentage of valid scans and framewise displacement. We chose the latter threshold given that a threshold of p = 0.005 uncorrected with minimum cluster size of 10 voxels (Lieberman & Cunningham, 2009) produces to spurious results (Eklund et al., 2016). Theferore, others have suggested (and recommended) a primary threshold of p < 0.001 (Woo et al., 2014). Also, in this study, we compared 4 groups instead of the usual two sample t-test, which could impact the power to detect significant differences using a standard FWE correction. It is also noteworthy to mention that using a more liberal thresholding (even uncorrected data) is encouraged to facilitate meta-analysis in neuroimaging (see (Salimi-Khorshidi et al., 2009). We also tested group differences in several region of interests (6 mm sphere) given our hypotheses: vmPFC (x = 0, y = 46, z=-10), vlPFC/aINS (left: x=-30, y = 22, z = 0; right: x = 36, y = 22, z=-4), and aMCC/pre-SMA (x = 8, y = 24, z = 36). Statistical threshold for ROI was determined using small volume correction pFWE < 0.05. Furthermore, we ran Levene’s tests given that unequal sample sizes may violate assumption regarding homogeneity of variances in F-test. Pairwise comparisons were conducted with Dunn’s Bonferroni correction for multiple comparisons. We then conducted non-parametric analysis of covariance (Quade’s tests) to examine the confounding effect of the severity of CP and ADHD symptoms.

## Results

### Identifying Variants Using ICU Total Score and Anxiety

#### Latent Profile Analysis

From the 1416 participants, 1315 had available data on at least one of the two variables of interests. Goodness of fit, as measured by AIC, BIC and SSA-BIC, revealed that the most significant decrease was observed moving from the 2-class to the 3-class solution. Moreover, the 3-class solution showed greater entropy than did the 4-class solution (Supplementary Tables 1 and Supplementary Fig. 1). The lowest group AvePP was higher than 0.80 and the lowest class size was > 1%. These three groups were: anxious group (ANX, 17.6% of the total sample), typically developing (TD, 79% of the total sample), and High ANX/CU+ (3.4% of the total sample) (Supplementary Fig. 2). Groups did not differ on age, sex, sites and motion parameters (Table 2). Unexpectedly, CU/ANX + group showed higher levels of CU traits, anxiety, and CP compared to the other groups. Both CU/ANX + and ANX showed higher levels of inattention symptoms and negative life events than did the TD group but did not statistically differ from each other.

**Table 2:** Sociodemographic and clinical differences between subclasses using ICU Total Score and SCARED

Characteristics	ANX (n = 231)	TD (n = 1039)	CU/ANX+ (n = 45)	Statistics	P value	Posthoc Between groups
Age	11.37 (3.19)	10.97 (3.38)	12.07 (3.69)	7.03	0.030	n.s.
Sex (Boys, %)	134 (58%)	658 (63.3%)	24 (53%)	3.78	0.151	
Race						
White/Caucasian	107 (51.7%)	456 (47.9%)	15 (39.5%)	18.12	0.053	n.s.
Black/African American	28 (13.5%)	161 (16.9%)	7 (18.4%)			
Hispanic	14 (6.8%)	116 (12.2%)	8 (21.1%)			
Asian	12 (5.8%)	26 (2.7%)	2 (5.3%)			
Other Races	4 (1.9%)	30 (3.2%)	0 (0%)			
Two or more Races	42 (20.3%)	163 (17.1%)	6 (15.8%)			
Sites				7.73	0.102	
Staten Island	42 (18.2%)	201 (19.3%)	14 (31.1%)			
RUBIC	110 (47.6%)	470 (45.2%)	23 (51.1%)			
CBIC	79 (34.2%)	368 (35.2%)	8 (17.8%)			
Valid Scans (%)	88.15%%	86.99%	88.71%	1.1	0.576	
Framewise Displ.	0.29 (0.38)	0.34 (0.44)	0.29 (0.42)	2.68	0.262	
*Callous-Unemotional Traits*						
ICU Total Score	25.2 (11.01)	23.66 (10.39)	31.15 (10.70)	647.44	< 0.001	CU/ANX > ANX, TD
Callousness	5.75 (4.35)	4.93 (3.97)	8.23 (5.26)	15.78	< 0.001	CU/ANX > TD
Uncaring	11.76 (5.23)	11.81 (4.92)	13.85 (5.42)	3.78	0.151	
Unemotional	5.81 (3.37)	4.84 (3.05)	7.54 (3.60)	25.73	< 0.001	CU/ANX > ANX > TD
Clinical Levels (%)						
Empirical Cut-Off (≥ 29)	25.30%	20.80%	46.20%	10.35	0.006	
90th percentile	12.70%	10.00%	34.60%	15.82	< 0.001	
*Anxiety*						
SCARED-P Raw Score	29.28 (5.34)	9.54 (6.13)	50.89 (7.09)	12.85	0.002	CU/ANX > ANX, TD
Clinical Levels (%)	78.40%	0%	100%	969.62	< 0.001	
*Conduct Problems*						
CBCL Raw Score	13.78 (9.96)	9.46 (9.32)	20.35 (12.45)	72.65	< 0.001	CU/ANX > ANX > TD
Clinical Levels (%)						
Empirical Cut-Off (≥ 65)	35.50%	19.10%	60.00%	58.07	< 0.001	
90th percentile	15.00%	8.20%	27.50%	23.48	< 0.001	
*ADHD symptoms*						
Hyperactivity/Imp.	0.43 (1.14)	0.15 (1.16)	0.42 (1.08)	15.31	< 0.001	ANX > TD
Inattention	0.98 (1.15)	0.57 (1.17)	1.12 (1.18)	35.78	< 0.001	CU/ANX, ANX > TD
*Adverse Childhood Events*						
Negative Life Events	7.19 (3.68)	6.15 (3.25)	7.9 (3.15)	24.89	< 0.001	CU/ANX, ANX > TD

*Note.* RUBIC = Rutgers University Brain Imaging Center; CBIC = CitiGroup Cornell Brain Imaging Center. Framewise displacement is calculated with ART’s composite motion FD measure. Chi-squared values were reported for analyses on categorical data; Kruskal-Wallis H values were reported for analyses on continuous measures. Posthoc analyses was tested using Dunn-Bonferroni correction p < 0.05)

#### Differences in Nucleus Accumbens Connectivity

**Fig. 1:**
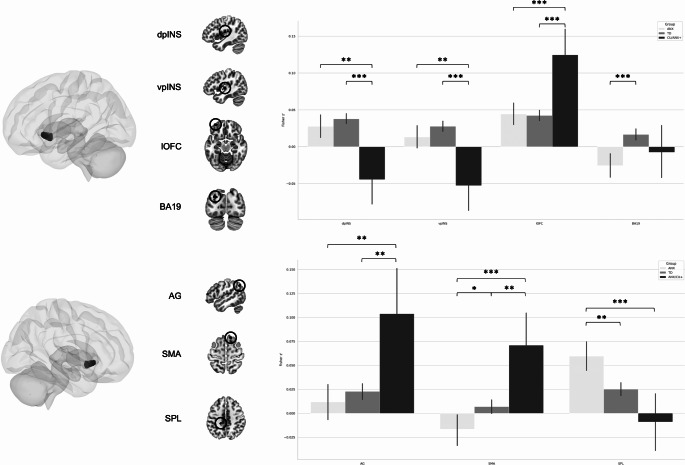
Bar graph representing group differences in Nucleus Accumbens’ functional connectivity (ICU Total Score – SCARED) (see also Table 3). dpINS = dorsal posterior Insula (probabilities of gray matter [GM], white matter [WM] and cerebrospinal fluid [CSF] are 0.80, 0.07, and 0.13 respectively); vpINS = ventral posterior insula (probabilities of GM, WM, and CSF are 0.61, 0.01, and 0.38, respectively); lOFC = lateral orbitofrontal cortex (probabilities of GM, WM, and CSF are 0.82, 0.03, and 0.07, respectively); BA19 = Brodmann Area 19 (probabilities of GM, WM, and CSF are 0.71, 0.02, and 0.27, respectively); AG = Angular Gyrus (probabilities of GM, WM, and CSF are 0.82, 0.13, and 0.02, respectively); SMA = Supplementary Motor Area (probabilities of GM, WM, and CSF are 0.67, 0.00, and 0.33, respectively); SPL = superior parietal lobule (5Ci) (probabilities of GM, WM, and CSF are 0.56, 0.39, and 0.05, respectively). ANX = Anxious group; TD = Typically Developing; CU/ANX + = High levels of CU & ANX. Pairwise comparisons were corrected using Dunn-Bonferroni. * p < 0.05; ** p < 0.01; *** p < 0.001

Comparing these three groups on (left and right) NAcc functional connectivity yield no significant differences when using a Family-wise correction threshold of p < 0.05. However, when using a more liberal threshold at a cluster level (> 20 voxels), differences in functional connectivity between the left NAcc and the left ventral (x=-44, y=-16, z = 2, F_(2, 1306)_ = 10.85, 31 voxels) and dorsal pINS (x=-44, y=-16, z = 18, F_(2, 1306)_ = 9.87, 41 voxels) were found (Fig. 1; Table 3). Group differences also revealed differences in functional connectivity between the left NAcc and the left lateral OFC (x=-32, y = 52, z=-14, F_(2, 1306)_ = 11.12, 38 voxels), and Brodmann Area 19 (x=-44, y=-16, z = 18, F_(2, 1306)_ = 9.87, 40 voxels). Posthoc indicated that for both ventral and dorsal pINS cluster, CU/ANX + showed greater negative coupling with the left NAcc, compared to ANX (*p* < 0.002 & *p* = 0.001, respectively) and TD (*ps* < 0.001). The CU/ANX + group also showed greater connectivity between the left NAcc and the lateral OFC compared to TD (p < 0.001), and ANX (p < 0.001). Finally, the ANX group showed greater negative coupling with Brodmann Area 19 than did TD (p < 0.001).

**Table 3:** Main differences in NAcc-to-voxel connectivity between subclasses (ICU Total Score + ANX)

Seeds (Targets)	Subclasses			*Post Hoc* ^A^
	G1: Moderate ANX/CU (17.6%)	G2: Low ANX / Moderate CU (79%)	G3: High ANX/ICU (3.4%)	
**L NACC**				
dorsal pINS	0.03 ± 0.12	0.04 ± 0.12	− 0.05 ± 0.11	G3 < G1, G2
Brodmann Area 19	− 0.03 ± 0.13	0.02 ± 0.13	− 0.01 ± 0.12	G1 < G2
lateral OFC	0.04 ± 0.12	0.04 ± 0.12	0.12 ± 0.12	G3 > G1, G2
ventral pINS	0.01 ± 0.12	0.03 ± 0.12	− 0.05 ± 0.11	G3 < G1, G2
**R NACC**				
Angular Gyrus	0.01 ± 0.14	0.02 ± 0.13	0.10 ± 0.16	G3 > G1, G2
SPL	0.06 ± 0.11	0.03 ± 0.12	− 0.01 ± 0.10	G1 > G2, G3
SMA	− 0.02 ± 0.13	0.01 ± 0.12	0.07 ± 0.12	G3 > G2 > G1

Note.Threshold p < 0.001 with 20 voxel extent. Dunn-Bonferroni post hoc correction (p < 0.05)

Group differences were also observed between the right NAcc and the SMA (x = 14, y = 26, z = 64, F_(2, 1306)_ = 10.33, 21 voxels), the left angular gyrus (x=-50, y=-64, z = 38, F_(2, 1306)_ = 9.55, 34 voxels), and the superior parietal lobule (SPL, 5Ci, x=-16, y=-32, z = 44, F_(2, 1306)_ = 11.34, 27 voxels) (Fig. 1; Table 3). Posthoc analyses revealed that the SMA result was driven by aberrant connectivity (not significant in TD, see Supplementary Table 2) in the CU/ANX + group compared to TD (p = 0.002), and ANX (p < 0.001), which significantly differed from each other (p = 0.013). The CU/ANX + also showed greater connectivity between the right NAcc and the angular gyrus, compared to TD (p = 0.006), and ANX (p = 0.002). Finally, the ANX group demonstrated greater connectivity with the SPL compared to TD (p = 0.002) and CU/ANX+ (p < 0.001). These results remained statistically significant after accounting for severity of CP and ADHD symptoms (*ps* < 0.001). Groups did not statistically differ on NAcc-to-predetermined ROIs.

### Identifying Subgroups Based on Callousness and Anxiety

#### Latent Profile Analysis

Comparing the 2 to 5-class models based on our criteria, we observed an elbow in AIC, BIC and SSA-BIC at the 4-class solution. The model yields good entropy (0.878), acceptable lowest class AvePP (> 0.80) and the smallest class size was higher than 1% (i.e., 3.5%) (See Supplementary Tables 3 and Supplementary Fig. 3). The identified classes were as followed: Anxious (ANX, 12.5%), typically developing (TD, 73.6%), primary variant (P1, 10.4%) and secondary variant (P2, 3.5%) (see Supplementary Fig. 4). Unsurprisingly, P1 and P2 showed higher callousness scores than Anxious and TD youths, whereas Anxious and P2 exhibited higher anxiety levels than the two other groups (Supplementary Table 4). Moreover, these four groups did not differ in terms of age, sex, sites, percentage of valid scans and movement parameters (Ps > 0.146). Moreover, although both variants showed higher levels of CU traits, conduct problems, hyperactivity/impulsivity and inattention symptoms compared to the other groups, they did not differ from each other.

#### Differences in Nucleus Accumbens Connectivity

Comparing these four groups on (left and right) NAcc functional connectivity yield no significant differences when using a Family-wise correction threshold of p < 0.05.

**Fig. 2:**
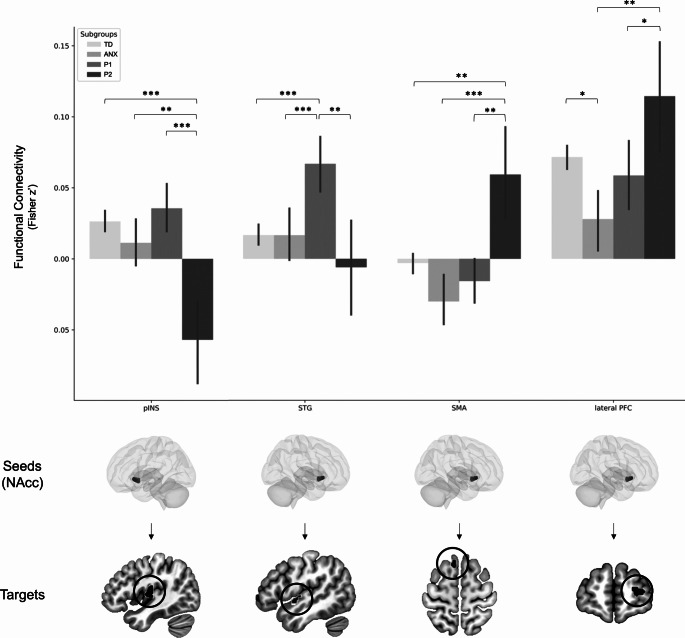
Bar graph representing group differences in Nucleus Accumbens’ functional connectivity (see also Supplementary Table 5). pINS = posterior Insula (probabilities of gray matter [GM], white matter [WM] and cerebrospinal fluid [CSF] are 0.70, 0.01, and 0.29 respectively); STG = Superior Temporal Gyrus (probabilities of GM, WM, and CSF are 0.68, 0.00, and 0.32, respectively); SMA = Supplementary Motor Area (probabilities of GM, WM, and CSF are 0.70, 0.25, and 0.05, respectively); lateral PFC = lateral Prefrontal Cortex (probabilities of GM, WM, and CSF are 0.76, 0.15, and 0.09, respectively). P1 = Primary Variant; P2 = Secondary Variant; ANX = Anxious group; TD = Typically Developing. Pairwise comparisons were corrected using Dunn-Bonferroni. * p < 0.05; ** p < 0.01; *** p < 0.001

Analyses with a more liberal threshold at a cluster level nonetheless revealed significant differences between the left NAcc and the left dorsal posterior insula (F_(3, 1306)_ = 9.03, x=-42, y=-16, z = 12, 103 voxels) as well as between the right NAcc and the superior temporal gyrus (STG) (F_(3, 1306)_ = 7.34, x=-48, y=-2, z=-6, 21 voxels), supplementary motor area (SMA) (F_(3, 1306)_ = 7.21, x=-8, y = 20, z = 60, 30 voxels) and lateral PFC (F_(3, 1306)_ = 6.53, x = 24, y = 60, z = 10, 47 voxels) (Fig. 2, Supplementary Table 5). Furthermore, including covariates as well as ADHD symptoms did not alter differences between subclasses and functional brain connectivity (ps < 0.001). However, adding the severity of CP as a covariate altered differences between subclasses regarding the NAcc – lateral PFC, but still remained statistically significant (F_(3, 1256)_ = 4.86, p = 0.002).Posthoc analyses revealed that the Secondary variant group showed significant decreased connectivity between the left NAcc and the left dorsal posterior insula, compared to the Anxious group (p = 0.003), TD group (p < 0.001) and the Primary variant group (p < 0.001), which did not significantly differ from each other. Furthermore, adolescents from the Primary variant group were characterized by increased connectivity between the right NAcc and the STG, in comparison to their counterparts from the Secondary variant group (p = 0.007), Anxious group (p = 0.001) and TD (p < 0.001). The Secondary variant group showed aberrant functional connectivity (not significant in the TD group, see Supplementary Table 6) between the right NAcc and the SMA in contrast to the Anxious group (p < 0.001), Primary variant group (p = 0.001) as well as TD (p = 0.005), whereas the Anxious group showed significant decreased connectivity between these regions compared to TD (p = 0.008). Finally, adolescents from the Secondary variant showed greater connectivity between the NAcc and the lateral PFC than their counterparts in the Anxious Group (p = 0.002), and Primary variant (p = 0.036) but not TD. Anxious group rather showed weaker connectivity between these regions than did TD (p = 0.011). Groups did not statistically differ on NAcc-to-predetermined ROIs.

## Discussion

In our study, we aimed to investigate differences between variants of psychopathy in NAcc functional brain connectivity using a large sample size of adolescents. Latent Profile Analysis using CU traits and anxiety revealed 3 homogeneous subclasses (ANX, TD, CU/ANX+) but failed to identify the expected variants. These groups did not statistically differ on functional connectivity of the NAcc when using a stringent statistical threshold across the whole-brain (p < 0.001 uncorrected with pFWE < 0.05). However, when using a more liberal threshold at a cluster level (> 20 voxels), we observed that groups differed on NAcc connectivity to the pINS, lOFC, BA19 as well as AG, SMA, and SPL. Secondary analyses using only the Callousness subscale of the ICU successfully identified the primary and the secondary variants. However, the four groups only statistically differed in NAcc functional connectivity when using a more liberal threshold (> 20 voxels), replicating the pINS and SMA findings and additionally showing a potential specific dysconnectivity between the NAcc and the STG in the primary variant. These results highlight the importance of studying subgroups of children and adolescents exhibiting high levels of callousness and offer novel insight about the potential neurobiological differences between variants.

Despite that individuals with high psychopathic traits are traditionally characterized by an absence of anxiety (Cleckley, 1951; Karpman, 1941) and fearlessness (Lykken, 1995), a non-negligible percentage of them actually report high levels of anxiety. Indeed, the secondary variant is thought to show a more severe clinical presentation compared to the prototypical one. In our data-driven analysis using the ICU total score and SCARED, we failed to identify the primary variant. While others have been unable to identify the primary (Euler et al., 2015; Lee et al., 2010) or the secondary variant (Colins et al., 2018; Goulter et al., 2017), one possible explanation is that the primary variant may be more easily identified through justice-involved sample including only males, whereas the secondary variant may be more prevalent in clinical settings including both sexes (Craig et al., 2021). Here, the community-referred recruitment model and the inclusion of both sexes may have explained the inability to find a primary variant across the sample. Also, of the data-driven studies aiming to identify variants of CU traits (Craig et al., 2021), majority uses other co-occurrent features (e.g., CP, physical, emotional, and/or sexual abuse, trauma) which raise the question whether the variants depend on other features rather than solely on levels of anxiety and CU traits. Still, some failed to identify the secondary variant even after adding other clustering features such as maltreatment and negative affect (Colins et al., 2018). Yet, another possibility is that some of the subconstructs of CU traits may blur the ability to adequately capture the inter-individual variability underpinning variants. In the current study, variants were successfully found when using the callousness score of the ICU, but not the total score. This may be partially explained by the fact that variance in subscales of the ICU may largely reflects variance from the general factor (Ray & Frick, 2020), but they remain only moderately correlated, as observed in the current study (*r* ranging from 0.40 to 0.66). Similarly, fear and anxiety are poorly distinguished in research on psychopathy (Hofmann et al., 2021; Hoppenbrouwers et al., 2016), leaving unknown whether deficits in threat detection or responsivity may improve the identification of variants compared to the usual subjective measure of trait anxiety. Unequivocally, future studies should specifically aim to identify the core features (the most optimal set of clustering variables) delineating the primary and secondary variants in order to provide a more standardized way to identify these children in research but also in clinical practice.

Individuals with co-occurrent psychopathic traits and high levels of anxiety are typically characterized by a dysregulated clinical profile which include borderline personality features (Blackburn & Coid, 1999; Goulter et al., 2019; Skeem et al., 2003, 2007). On a neurobiological level, we found that this particular group significantly differ from TD, and ANX, in functional connectivity between the NAcc and pINS, lOFC, AG, and SMA. However, when comparing the secondary to the primary variants (found in the subsequent analyses), only the NAcc-pINS and NAcc-SMA connectivity replicated, suggesting important deficits in the secondary group. In the TD group, we found a significant connectivity between the NAcc and the pINS, but not with the SMA, suggesting that the latter connectivity may be aberrant in the secondary variant. While the interpretation of this aberrant connectivity remain elusive, further investigation is necessary to identify whether this functional connectivity may be related to specific symptoms not found in TD, may reflect a brain reorganization, or a spurious result. Across neuroimaging literature, the pINS appear to be implicated in processing sensory information (i.e., interoceptive processes, (Kurth et al., 2010; Tian & Zalesky, 2018), whereas the SMA is often linked to motor planning, sensory and memory tasks (Chung et al., 2005; Sheets et al., 2021), future studies should aim to examine the functional roles of these connectivity in the specific symptomatology of children with the secondary variant that may distinguish them from the primary variant.

Prior work suggested that individuals with the primary variant may be characterized by abnormal decision-making including utilitarian moral decision (Koenigs et al., 2010, 2012). We thus hypothesized that this group may be characterized by decreased functional connectivity between the NAcc and vmPFC. However, groups did not significantly differ in NAcc-vmPFC connectivity. However, we found an increased connectivity between NAcc and STG in the primary variant compared to other groups. In healthy subjects, both NAcc and STG are co-activated during reward processing (Arsalidou et al., 2020; Lopez-Gamundi et al., 2021; Wilson et al., 2018) but also during social cognition including self-agency (Sperduti et al., 2011) and personal perspective during moral reasoning (Boccia et al., 2017). Deficits in activity of this particular region was observed in offenders with an antisocial personality disorder and psychopathy during reversal learning (i.e., rewarded responses > punished errors) (Gregory et al., 2015). Although we did not find any difference in functional connectivity between the core regions of reward processing, the NAcc-STG connectivity highlights the interaction between reward and other potential networks (e.g., social cognition) that may underpin behaviors that are specific to the primary variants.

## Limitations

The current study aimed to examine differences in NAcc functional connectivity between variants of callous traits using a large sample of children and adolescents. Nevertheless, some limitations need to be acknowledged. First, our sample comprised children and adolescents with psychopathologies, recruited using a community-referred recruitment model. It is thus difficult to interpret our results given the absence of a true control group with no psychopathologies. Furthermore, the absence of such group could have reduced the ability to detect significant differences between groups. We still found significant between-group differences using a large sample. Studies should seek to examine whether the functional connectivity differences found in our study significantly discriminate between variants of callous traits and healthy controls. Secondly, neuroimaging suffers from a replicability crisis, which increase concerns about spurious results due to limited sample size and methodologies. In our study, the length of resting-state fMRI was relatively short (10 min). However, the UK Biobank include only 6 min resting-state scanning session and show similar results to those with > 20 min (i.e., ABCD & HCP) (Marek et al., 2022). A longer scanning session from 10 to 20 min (Anderson et al., 2011; Birn et al., 2013; O’Connor et al., 2017) is then preferred to a single 5-6 min, however gains in intersession reliability reduce after 9–12 min (Birn et al., 2013). In addition to the scan length (i.e., 10 min total), the Healthy Brain Network include a TR = 0.8 with a multiband of 6, which inherently increases the number of acquired volumes (see (Jahanian et al., 2019; Liao et al., 2013). We acknowledge that a longer scanning session would have been optimal, the scan length, the sample size and the number of volumes acquired meet the current recommendations in the resting-state neuroimaging literature; suggesting that they should provide reliable estimates. Studies aiming to replicate our findings are strongly encouraged. Thirdly, prior work using data-driven techniques to identify variants with the ICU also include other variables such as childhood maltreatment (Craig et al., 2021). In our study, childhood maltreatment was not assessed. Since adverse childhood events are not equivalent to childhood maltreatment, it remains unknown whether the secondary variant found in our study reported higher childhood maltreatment compared to the primary variant group. Lastly, we did not use IQ as a potential confounder given that theoretical framework of the brain structures underpinning IQ does not involve the NAcc (Jung & Haier, 2007). However, it is possible that including IQ as a covariate may have provided a more precise estimate of the NAcc-lateral PFC.

## Conclusion

In the current study, we were able to identify alterations in NAcc connectivity in clinically relevant subgroups of children with severe levels of callousness, using a large sample of adolescents. While adolescents with the primary variant were characterized by increased functional connectivity between the NAcc and a brain region involved in first perspective moral reasoning and decision-making (i.e., STG), their counterparts with the secondary variant displayed reduced functional connectivity between the NAcc and an interoceptive region (i.e., pINS) as well as increased connectivity between NAcc and brain regions involved in cognitive control (i.e., SMA, lPFC). In the future, longitudinal studies will be warranted to better understand the actual development of the functional connectivity described here, as well as to clarify whether they relate to variants in adults.

### Electronic Supplementary Material

Below is the link to the electronic supplementary material.

Supplementary Material 1
