# Supplementary material for: Functional Connectivity of the Nucleus Accumbens across Variants of Callous-Unemotional Traits: A Resting-State fMRI Study in Children and Adolescents
Source: Res Child Adolesc Psychopathol. 2023 Oct 25;52(3):353–68. doi: 10.1007/s10802-023-01143-z (PMC10896801; doi:10.1007/s10802-023-01143-z)
Supplement: Supplementary file 1 — Supplementary Material 1 [file 10802_2023_1143_MOESM1_ESM.docx]

– SUPPLEMENTARY MATERIAL –

**Functional Connectivity of the Nucleus Accumbens across Variants of Callous-Unemotional Traits: a Resting-State fMRI Study in Children and Adolescents**

Jules R. Dugré, PhD ^1^, & Stéphane Potvin, PhD ^2,3^

^1^ School of Psychology and Centre for Human Brain Health, University of Birmingham,

Birmingham

^2^ Research Center of the Institut Universitaire en Santé Mentale de Montréal, Montreal, Canada

^3^ Department of Psychiatry and Addictology, Faculty of medicine, University of Montreal, Montreal, Canada

**Corresponding authors**

Jules Roger Dugré, PhD

Centre for Human Brain Health, University of Birmingham, School of Psychology, Birmingham B15 2TT

Email: j.dugre@bham.ac.uk.

&

Stéphane Potvin, PhD;

Centre de recherche de l’Institut Universitaire en Santé Mentale de Montréal; 7331 Hochelaga; Montreal, Canada; H1N 3V2;

Email: [stephane.potvin@umontreal.ca](mailto:stephane.potvin@umontreal.ca)

Contents

[**Supplementary Table 1.** Latent Profile Analysis for 2- to 5-class solutions (ICU Total Score + SCARED) 3](#_Toc141965432)

[**Supplementary Figure 1.** Results of the LPA (ICU-Total+SCARED). 4](#_Toc141965433)

[**Supplementary Figure 2.** Distribution of the Latent Profile Analysis (ICU-Total & SCARED). 5](#_Toc141965434)

[**Supplementary Table 2.** One-Sample t-test within the TD group (ICU-Total + SCARED) 6](#_Toc141965435)

[**Supplementary Table 3.** Latent Profile Analysis for 2- to 5-class solutions (ICU-C & SCARED) 7](#_Toc141965436)

[**Supplementary Figure 3.** Results of the LPA (ICU-Callousness+SCARED). 8](#_Toc141965437)

[**Supplementary Figure 4.** Distribution of the Latent Profile Analysis (ICU-C & SCARED) 9](#_Toc141965438)

[**Supplementary Table 4.** Sociodemographic and clinical differences between subclasses (ICU-C & SCARED) 10](#_Toc141965439)

[**Supplementary Table 5.** Main differences in NAcc-to-voxel connectivity between subclasses (ICU-C & SCARED) 11](#_Toc141965440)

[**Supplementary Table 6.** One-Sample t-test within the TD group (ICU-C + SCARED) 12](#_Toc141965441)

| **Supplementary Table 1.** Latent Profile Analysis for 2- to 5-class solutions (ICU Total Score + SCARED) | | | | |  |
| --- | --- | --- | --- | --- | --- |
| Metrics | LPA solutions | | | |  |
|  | 2-class | 3-class | 4-class | 5-class |  |
| AIC | 20463.32 | **20378.33** | 20337.09 | 20299.84 |  |
| BIC | 20561.77 | **20492.33** | 20466.64 | 20444.92 |  |
| SSA BIC | 20501.41 | **20422.44** | 20387.22 | 20355.98 |  |
| lowest class | 12.17% | **3.70%** | 3.10% | 1.18% |  |
| entropy | 0.953 | **0.923** | 0.887 | 0.901 |  |
| lowest AvePP | 0.879 | **0.858** | 0.813 | 0.768 |  |
| *Note. AIC = Akaike Information Criteria; BIC = Bayesian Information Criteria; SSA BIC = Sample-size adjusted BIC; AvePP = Average Posterior Probabilities.* | | | | |  |
|  |  |  |  |  |  |

**Supplementary Figure 1.** Results of the LPA (ICU-Total+SCARED). Results of the LPA for 2- to 5-class solutions revealed that the 3-class solution was the most optimal for the ICU total score and SCARED-P. AIC = Akaike Information Criteria; BIC = Bayesian Information Criteria; SSA BIC = Sample-size adjusted BIC.


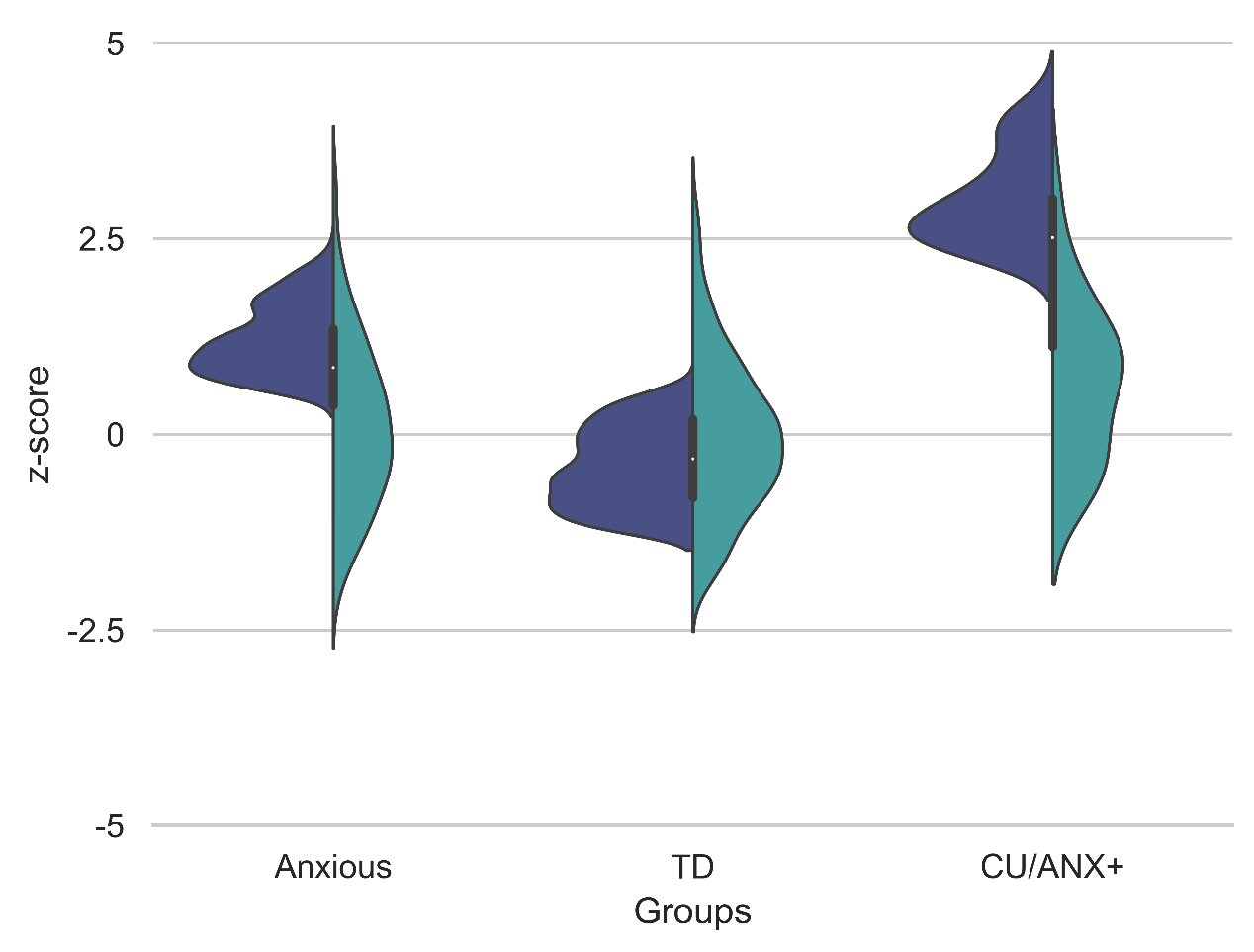


**Supplementary Figure 2.** Distribution of the Latent Profile Analysis (ICU-Total & SCARED). Anxiety=Blue; CU traits=teal; ANX = Anxious subjects; TD = Typically Developing; CU/ANX+ = High on both Callous-unemotional traits & Anxiety.

| **Supplementary Table 2.** One-Sample t-test within the TD group (ICU-Total + SCARED) | |
| --- | --- |
| **Seeds** (Targets) | t-value |
|  |  |
| **L NACC** |  |
| dorsal pINS | 11.34* |
| Brodmann Area 19 | 7.37* |
| lateral OFC | 12.44* |
| ventral pINS | 9.92* |
| ROIs |  |
| vmPFC | 47.38* |
| right FIC | 21.25* |
| left FIC | 34.51* |
| right dlPFC | 14.84* |
| left dlPFC | 20.31* |
| aMCC | 34.15* |
| **R NACC** |  |
| Angular Gyrus | 8.42* |
| SMA | 1.58 |
| SPL | 9.2* |
| ROIs |  |
| vmPFC | 44.27* |
| right FIC | 18.64* |
| left FIC | 27.91* |
| right dlPFC | 17.14* |
| left dlPFC | 14.17* |
| aMCC | 26.99* |
| Note. *Threshold pFWE<0.05 (svc.) | |

| **Supplementary Table 3.** Latent Profile Analysis for 2- to 5-class solutions (ICU-C & SCARED) | | | | |  |
| --- | --- | --- | --- | --- | --- |
| Metrics | LPA solutions | | | |  |
|  | 2-class | 3-class | **4-class** | 5-class |  |
| AIC | 18671.64 | 18565.464 | **18492.37** | 18450.78 |  |
| BIC | 18770.042 | 18679.39 | **18621.83** | 18595.78 |  |
| SSA BIC | 18709.69 | 18609.51 | **18542.42** | 18506.83 |  |
| lowest class | 11.99% | 9.68% | **3.50%** | 3.20% |  |
| entropy | 0.955 | 0.898 | **0.878** | 0.872 |  |
| lowest AvePP | 0.877 | 0.837 | **0.8** | 0.779 |  |
| *Note. AIC = Akaike Information Criteria; BIC = Bayesian Information Criteria; SSA BIC = Sample-size adjusted BIC; AvePP = Average Posterior Probabilities.* | | | | |  |
|  |  |  |  |  |  |


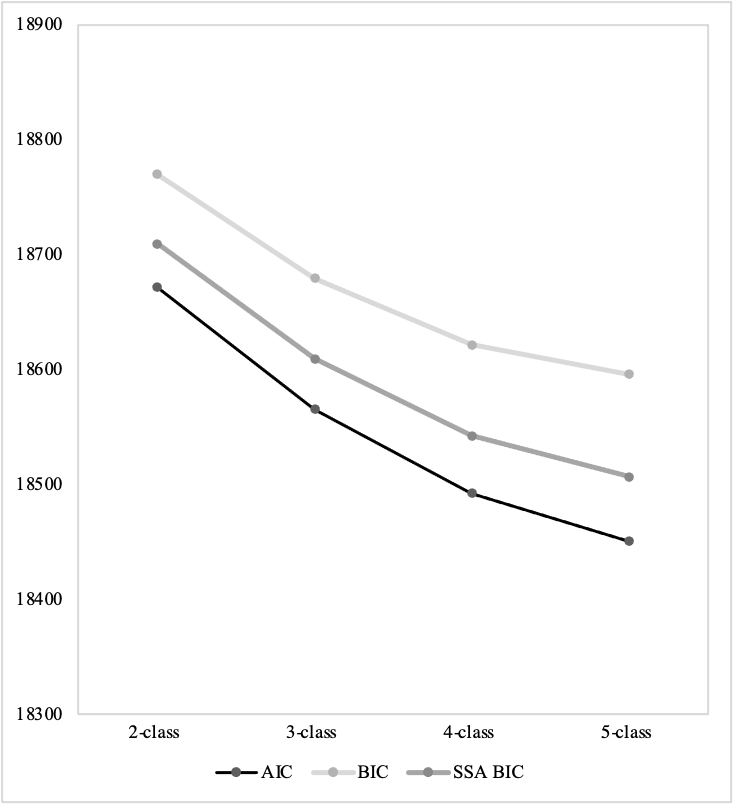


**Supplementary Figure 3.** Results of the LPA (ICU-C+SCARED). Results of the LPA for 2- to 5-class solutions revealed that the 4-class solution was the most optimal based on the AIC, BIC and SSA BIC. AIC = Akaike Information Criteria; BIC = Bayesian Information Criteria; SSA BIC = Sample-size adjusted BIC.


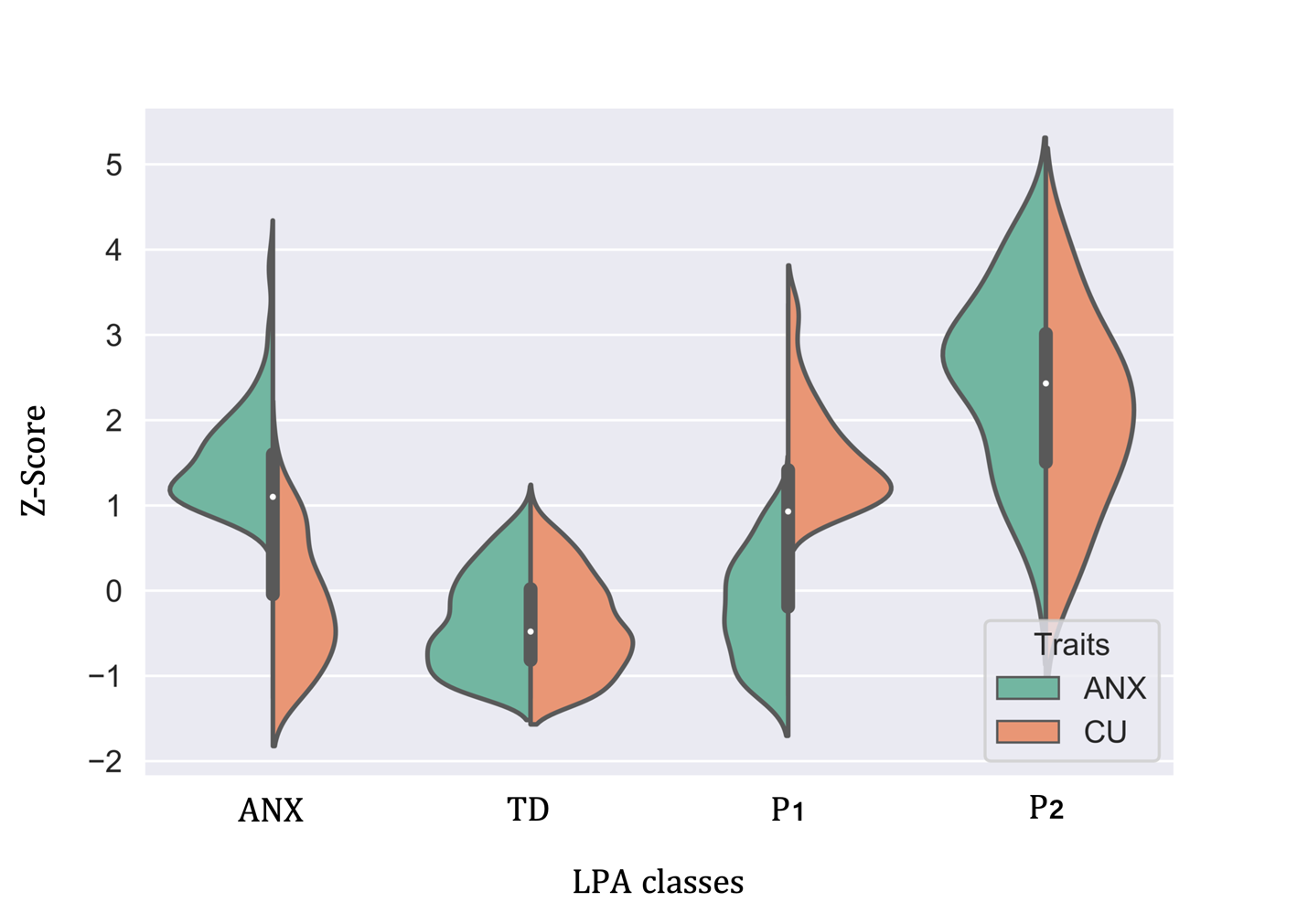


**Supplementary Figure 4.** Distribution of the Latent Profile Analysis (ICU-C & SCARED). ANX = Anxious subjects; TD = Typically Developing; P1 = Primary Variant Callousness; P2 = Secondary Variant of Callousness

| **Supplementary Table 4.** Sociodemographic and clinical differences between subclasses (ICU-C & SCARED) | | | | | | | |
| --- | --- | --- | --- | --- | --- | --- | --- |
| Characteristics | Anxious (N=164) | TD  (N=965) | P1 Variant (N=136) | P2 Variant (N=46) | Statistics | P value | Posthoc Between groups |
|  |  |  |  |  |  |  |  |
| Age | 11.5 (3.3) | 10.9 (3.4) | 10.9 (3.4) | 11.9 (3.6) | 5.4 | 0.147 | - |
| Sex (Boys, %) | 89 (54.3%) | 607 (62.9%) | 89 (65.4%) | 30 (65.2%) | 5.4 | 0.146 | - |
| Race |  |  |  |  |  |  |  |
| White/Caucasian | 74 (50.7%) | 425 (48.0%) | 62 (48.8%) | 17 (44.7%) | 17.46 | 0.292 |  |
| Black/African American | 23 (15.8%) | 139 (15.7%) | 29 (22.8%) | 4 (10.5%) |  |  |  |
| Hispanic | 11 (7.5%) | 112 (12.7%) | 7.1 (9.0%) | 6 (15.8%) |  |  |  |
| Asian | 8 (5.5%) | 25 (2.8%) | 4 (3.1%) | 3 (7.9%) |  |  |  |
| Other Races | 2 (1.4%) | 28 (3.2%) | 3 (2.4%) | 1 (2.6%) |  |  |  |
| Two or more Races | 28 (19.2%) | 156 (17.6%) | 20 (15.7%) | 7 (18.4%) |  |  |  |
| Sites |  |  |  |  |  |  |  |
| Staten Island | 38 (23.2%) | 186 (19.3%) | 19 (14%) | 11 (23.9%) | 10.4 | 0.111 | - |
| RUBIC | 72 (43.9%) | 431 (44.7%) | 74 (54.4%) | 25 (54.3%) |  |  |  |
| CBIC | 54 (32.9%) | 348 (36.1%) | 43 (31.6%) | 10 (21.7%) |  |  |  |
|  |  |  |  |  |  |  |  |
| Valid Scans (%) | 88.6% | 87.2% | 85.8% | 87.1% | 2.2 | 0.525 | - |
| Framewise Displ. | .29 (.39) | .33 (.41) | .43 (.62) | .29 (.43) | 3.5 | 0.324 | - |
|  |  |  |  |  |  |  |  |
| *CU Traits - ICU* |  |  |  |  |  |  |  |
| ICU Total Score | 22.9 (9.01) | 20.6 (7.96) | 38.3 (7.42) | 40.2 (9.3) | 331.6 | <0.001 | P2, P1 > ANX, TD |
| Callousness | 4.59 (2.97) | 3.50 (2.33) | 11.65 (2.49) | 13.5 (4.5) | 407.6 | <0.001 | P1, P2 > ANX > TD |
| Uncaring | 10.94 (5.09) | 10.68 (4.35) | 17.1 (3.8) | 16.5 (4.4) | 211.5 | <0.001 | P2, P1 > ANX, TD |
| Unemotional | 5.73 (3.14) | 4.40 (2.82) | 7.09 (3.12) | 8.4 (4.2) | 102.5 | <0.001 | P2, P1 > ANX > TD |
|  |  |  |  |  |  |  |  |
| Clinical Levels (%) |  |  |  |  |  |  |  |
| Empirical Cut-Off (≥ 29) | 25.9% | 16.4% | 91.9% | 93.5% | 357.0 | <0.001 | - |
| 90th percentile | 5.6% | 0.1% | 49.3% | 61.3% | 364.5 | <0.001 | - |
|  |  |  |  |  |  |  |  |
| *Anxiety* |  |  |  |  |  |  |  |
| SCARED-P | 33.2 (6.9) | 10.25 (6.8) | 12.28 (7.5) | 44.9 (12.4) | 519.1 | <0.001 | P2, ANX > P1 > TD |
|  |  |  |  |  |  |  |  |
| *CP - CBCL* |  |  |  |  |  |  |  |
| Raw Score | 13.7 (9.7) | 8.0 (8.0) | 20.4 (10.1) | 23.3 (12.3) | 241.9 | <0.001 | P1, P2 > ANX > TD |
|  |  |  |  |  |  |  |  |
| Clinical Levels (%) |  |  |  |  |  |  |  |
| T-Score ≥ 65 | 36.3% | 14.2% | 59.2% | 63.4% | 189.9 | <0.001 | - |
| 90th percentile | 14.0% | 4.5% | 33.8% | 41.5% | 162.4 | <0.001 | - |
|  |  |  |  |  |  |  |  |
| *ADHD symptoms* |  |  |  |  |  |  |  |
| Hyperactivity/Imp. | .39 (1.1) | .08 (1.1) | .69 (1.2) | .73 (1.5) | 50.1 | <0.001 | P2, P1, ANX > TD |
| Inattention | .87 (1.2) | .48 (1.2) | 1.35 (.9) | 1.48 (.9) | 106.4 | <0.001 | P2, P1 > ANX > TD; |
|  |  |  |  |  |  |  |  |
| *Adverse Childhood Events* |  |  |  |  |  |  |  |
| Negative Life Events | 7.1 (3.67) | 6.1 (3.3) | 7.2 (3.4) | 7.7 (3.4) | 25.9 | <0.001 | P2, P1, ANX > TD |
| *Note.* RUBIC = Rutgers University Brain Imaging Center; CBIC = CitiGroup Cornell Brain Imaging Center. Framewise displacement is calculated with ART's composite motion FD measure. Chi-squared values were reported for analyses on categorical data; Kruskal-Wallis H values were reported for analyses on continuous measures. Posthoc analyses was tested using Dunn-Bonferroni correction p<0.05) | | | | | | | |
|  |  |  |  |  |  |  |  |

| **Supplementary Table 5.** Main differences in NAcc-to-voxel connectivity between subclasses (ICU-C & SCARED) | | | | | |  |
| --- | --- | --- | --- | --- | --- | --- |
| **Seeds** (Targets) | Subclasses | | | | *Post Hoc*A |  |
|  | ANX | TD | P1 | P2 |  |  |
| **L NACC** |  |  |  |  |  |  |
| L Posterior Insula | 0.011 ± 0.104 | 0.026 ± 0.107 | 0.036 ± 0.104 | -0.057 ± 0.097 | P2 < TD, ANX, P1 |  |
| **R NACC** |  |  |  |  |  |  |
| L STG/INS | 0.017 ± 0.124 | 0.017 ± 0.115 | 0.067 ± 0.112 | -0.006 ± 0.116 | P1 > TD, ANX, P2 |  |
| L SMA | -0.03 ± 0.110 | -0.003 ± 0.110 | -0.016 ± 0.098 | 0.059 ± 0.117 | P2 > TD, ANX, P1; TD > ANX |  |
| R Lateral PFC | 0.028 ± 0.134 | 0.072 ± 0.131 | 0.059 ± 0.149 | 0.115 ± 0.132 | TD, P2 > ANX; P2 > P1 |  |
| Note.Threshold p<0.001 with 20 voxel extent. Dunn-Bonferroni post hoc correction (p<0.05). | | | | | |  |
|  |  |  |  |  |  |  |

| **Supplementary Table 6.** One-Sample t-test within the TD group (ICU-C + SCARED) | |
| --- | --- |
| **Seeds** (Targets) | t-value |
|  |  |
| **L NACC** |  |
| pINS | 11.16* |
| ROIs |  |
| vmPFC | 44.11* |
| right FIC | 20.62* |
| left FIC | 32.73* |
| right dlPFC | 14.89* |
| left dlPFC | 20.44* |
| aMCC | 33.04* |
| **R NACC** |  |
| lOFC | 19.39* |
| SMA | 0.70 |
| STG | 7.54* |
| ROIs |  |
| vmPFC | 41.52* |
| right FIC | 18.5* |
| left FIC | 25.52* |
| right dlPFC | 17.61* |
| left dlPFC | 14.55* |
| aMCC | 26.88* |
| Note. *Threshold pFWE<0.05 (svc.) | |
